# Interactive analysis of biosurfactants in fruit-waste fermentation samples using BioSurfDB and MEGAN

**DOI:** 10.1038/s41598-022-11753-0

**Published:** 2022-05-11

**Authors:** Gabriela Fiori da Silva, Anupam Gautam, Iolanda Cristina Silveira Duarte, Tiago Palladino Delforno, Valéria Maia de Oliveira, Daniel H. Huson

**Affiliations:** 1 Department of Biology, Federal University of São Carlos, Rodovia João Leme dos Santos km 110 SP-264, Bairro Itinga, Sorocaba, SP 18052-780 Brazil; 2 Institute for Bioinformatics and Medical Informatics, University of Tübingen, Sand 14, 72076 Tübingen, Germany; 3 International Max Planck Research School “From Molecules to Organisms”, Max Planck Institute for Biology, Max-Planck-Ring 5, Tübingen, 72076 Germany; 4 Innovation Institute for Biotechnology (SENAI-Biotech), Rua Anhaia, 1321, Bom Retiro, SP 01130-000 Brazil; 5 Microbial Resources Division, Research Center for Chemistry, Biology and Agriculture, University of Campinas, Campinas, SP 13083-970 Brazil

**Keywords:** DNA sequencing, Functional clustering, Software, Metagenomics

## Abstract

Agroindustrial waste, such as fruit residues, are a renewable, abundant, low-cost, commonly-used carbon source. Biosurfactants are molecules of increasing interest due to their multifunctional properties, biodegradable nature and low toxicity, in comparison to synthetic surfactants. A better understanding of the associated microbial communities will aid prospecting for biosurfactant-producing microorganisms. In this study, six samples of fruit waste, from oranges, mangoes and mixed fruits, were subjected to autochthonous fermentation, so as to promote the growth of their associated microbiota, followed by short-read metagenomic sequencing. Using the DIAMOND+MEGAN analysis pipeline, taxonomic analysis shows that all six samples are dominated by Proteobacteria, in particular, a common core consisting of the genera *Klebsiella*, *Enterobacter*, *Stenotrophomonas*, *Acinetobacter* and *Escherichia*. Functional analysis indicates high similarity among samples and a significant number of reads map to genes that are involved in the biosynthesis of lipopeptide-class biosurfactants. Gene-centric analysis reveals *Klebsiella* as the main assignment for genes related to putisolvins biosynthesis. To simplify the interactive visualization and exploration of the surfactant-related genes in such samples, we have integrated the BiosurfDB classification into MEGAN and make this available. These results indicate that microbiota obtained from autochthonous fermentation have the genetic potential for biosynthesis of biosurfactants, suggesting that fruit wastes may provide a source of biosurfactant-producing microorganisms, with applications in the agricultural, chemical, food and pharmaceutical industries.

## Introduction

Biosurfactants are surface-active molecules produced by microorganisms that have been highlighted as an environmentally-friendly alternative to their synthetic counterpart, chemical surfactants^1,2^, which are produced by the petrochemical industry. Microbial surfactants demonstrate higher degradability, lower toxicity, selectivity, antimicrobial and anti-adhesive properties, and applicability in a large range of values of pH, temperature, and salinity^3–5^. They are versatile and have applications in pharmaceutics, food, cosmetics, agriculture, wastewater, bioremediation, enhanced oil recovery, metal removal and other industrial sectors^6–8^.

Biosurfactant-producing microorganisms have been reported and isolated from several sources, including marine habitats, mangroves, freshwater, soil, sludge and fruits^9–12^. In particular, fruit residues are important natural microhabitats for microorganisms due to their organic matter content, low pH and high sugar content, making them a source of diverse microorganisms^13^. Several studies have used microorganisms isolated from fruits in the production of various molecules of biotechnological interest, such as mannitol, hydrogen, organic acids, biofuels and biosurfactants^14–18^. In addition, fruit waste and residues generated by fruit-processing industries can also be used as a renewable and low-cost carbon source for fermentative processes^12,19–21^. To allow better exploitation of such waste as a source of useful microorganisms, a clearer understanding of the taxonomic and functional diversity of the present microbes is required. Shotgun metagenomic sequencing and subsequent alignment-based analysis allow one to identify specific genes of interest that are related to biosurfactant production.

Various metagenomic approaches combined with bioinformatics tools have been used to study biosurfactants^22^, such as AntiSMASH, designed for the identification, functional annotation, and analysis of biosynthetic gene clusters^23^. However, general-purpose functional databases, such as KEGG^24^, are not ideal for this specific purpose as they tend to focus on other aspects of function. In particular, some genes related to biosurfactant biosynthesis are classified under antibiotics in nonribosomal peptide pathways. Hence, the use of a domain-specific database is of advantage^25^.

Here we present six short-read metagenomic datasets that we have collected from different fruit autochthonous fermentation batch reactors using short-read Illumina sequencing. We used the DIAMOND+MEGAN^26^ pipeline to evaluate the microbial diversity present in the samples and to detect and analyze the genes involved in biosurfactant biosynthesis pathways. In more detail, taxonomy analysis was performed by MEGAN based on DIAMOND alignments against the NCBI-nr protein reference database^27^. For functional analysis, here we present a new extension of MEGAN that allows functional analysis based on the BiosurfDB database, a domain-specific database focused on identifying genes related to biosurfactant production and biodegradation^28^. To firmly establish the taxonomic identity of the organisms that contain the genes related to biosurfactant production, we applied gene-centric assembly^29^ to those genes and aligned the resulting gene-length contigs against the NCBI-nt database^27^ using BLASTN^30^.

Our analysis indicates that the six samples possess a common core of Gammaproteobacteria, dominated by the genera *Klebsiella*, *Enterobacter*, *Stenotrophomonas*, *Escherichia* and *Acinetobacter*. The samples have similar functional profiles and show a potential for biosurfactant biosynthesis, especially lipopeptides. The biosurfactant-related genes are predominately associated with *Klebsiella* sp. and *Enterobacter* sp., and, to a lower degree, with *Acinetobacter* sp. 89 and *Stenotrophomonas* sp.

## Results

### Taxonomic analysis

Each of the six metagenomic datasets was sequenced to over 20 million reads per sample, using Illumina sequencing in a 2 $\times$ 150 bp layout. Of 140, 183, 232 total input sequencing reads, 121, 875, 304 obtained at least one alignment against the NCBI-nr database, and of these, 121, 561, 304 could be assigned to a taxon in the NCBI taxonomy, leading to an assignment rate of $87\%$.

In all samples, reads were predominantly assigned to bacteria by an NCBI-nr run of the DIAMOND+MEGAN pipeline. The three main phyla are Proteobacteria followed by Firmicutes and Actinobacteria (Table 1). Rarefaction analysis (see Supplementary Figure S1) showed that the number of genera detected reached a plateau for each of the samples, indicating that the amount of sequencing performed was sufficient to obtain a stable representation of the taxonomic content of the samples. Rarefaction analysis suggests that the Mango and Mix samples have slightly higher diversity than the Orange samples, which is also reflected in the Shannon–Weaver diversity indices ($\alpha$-diversity measure) calculated from the genus-level assignments, which are 2.0, 1.6, 2.0, 1.9, 0.5 and 1.0, for the Mango-1, Mango-2, Mix-1, Mix-2, Orange-1 and Orange-2 samples, respectively. Further, a $\beta$-diversity analysis at genus-level (see Supplementary Figure S2) shows that all pairs of samples cluster with each other.

**Figure 1:**
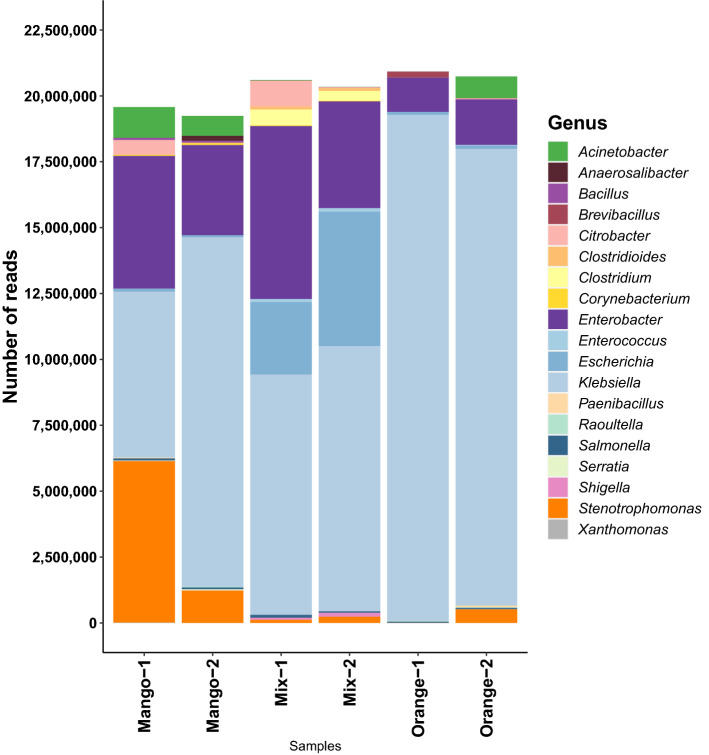
For each of the six fruit samples, we show the number of reads assigned to each of 19 occurring genera, based on an NCBI-nr run of the DIAMOND+MEGAN pipeline. Note that “taxonomic projection” was performed to map all read assignments to the genus level.

Most of the taxonomic content of the six samples falls into 19 genera (Fig. 1). *Klebsiella* dominates all six samples, accounting for 91.8 % and 83.6 % of genus-level assignments in the Orange-1 and Orange-2 samples respectively, and with assignment rates of 32.2 %, 68.9 %, 44.3 % and 49.5 %, for Mango-1, Mango-2, Mix-1 and Mix-2, respectively. Differences in the proportion of reads assigned to specific genera were observed between all six samples, which is to be expected even between the duplicates, as they are biological replicates (Table 2).

The NCBI-nr runs of MEGAN indicate that the six fruit samples studied here are dominated by Gammaproteobacteria, with most reads assigned to the genera *Klebsiella* ($32.2{-}91.8$ %, average 61.7 %), *Enterobacter* ($6.3{-}31.8$ %, average 18.3 %), *Stenotrophomonas* ($0.0{-}31.3$ %, average 7.0 %), *Escherichia* ($0.5{-}21.5$ %, average 6.8 %) and *Acinetobacter* ($0.0{-}6.0$ %, average 2.4 %). The first, second and fourth genera are members of the Enterobacteriaceae family, whereas the third and last belong to the family of Lysobacteriaceae and Moraxellaceae, respectively.

**Table 1:** For each of the six fruit samples, we report the total percentage of reads assigned to the bacterial domain and to three top bacterial phyla.

	Mango-1	Mango-2	Mix-1	Mix-2	Orange-1	Orange-2
**Domain**						
Bacteria	99.9	99.9	99.9	99.9	99.8	99.9
**Phylum**						
Proteobacteria	99.1	98.3	95.8	96.8	99.0	99.6
Firmicutes	0.6	1.3	4.0	3.1	1.0	0.4
Actinobacteria	0.2	0.4	0.1	0.1	0.0	0.0

**Table 2:** For each of the six fruit samples, we report the relative percentage of reads assigned to the six top bacterial genera.

	Mango-1	Mango-2	Mix-1	Mix-2	Orange-1	Orange-2
*Klebsiella*	32.2	68.9	44.3	49.5	91.8	83.6
*Enterobacter*	25.7	17.8	31.8	19.9	6.3	8.3
*Stenotrophomonas*	31.3	6.4	0.6	1.2	0.0	2.5
*Escherichia*	0.5	0.5	13.3	25.1	0.5	0.6
*Acinetobacter*	6.0	3.9	0.1	0.1	0.0	4.0
*Citrobacter*	2.9	0.1	4.8	0.2	0.0	0.2

### Functional analysis

**Figure 2:**
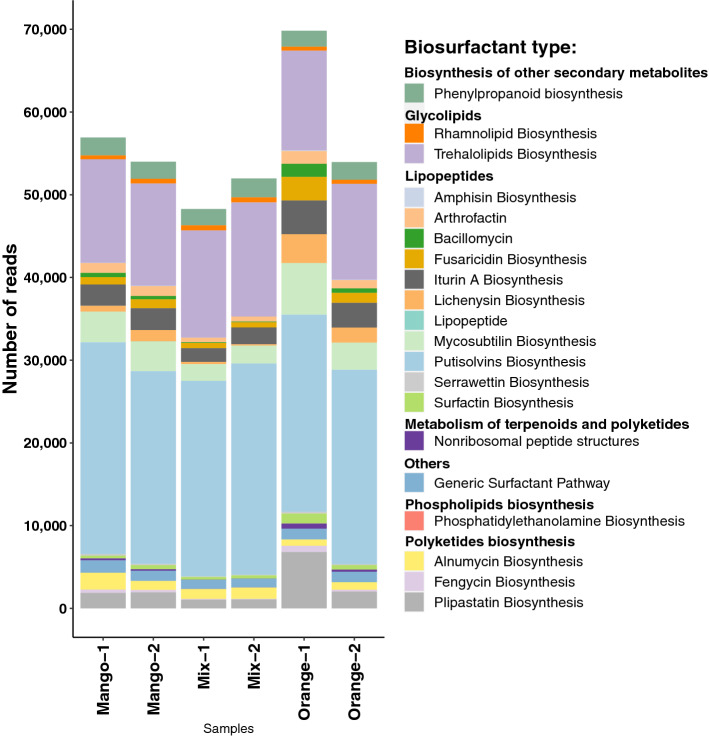
For each of the six fruit samples, we report the number of reads that are assigned to a surfactant class, based on a BioSurfDB run of the DIAMOND+MEGAN pipeline.

**Figure 3:**
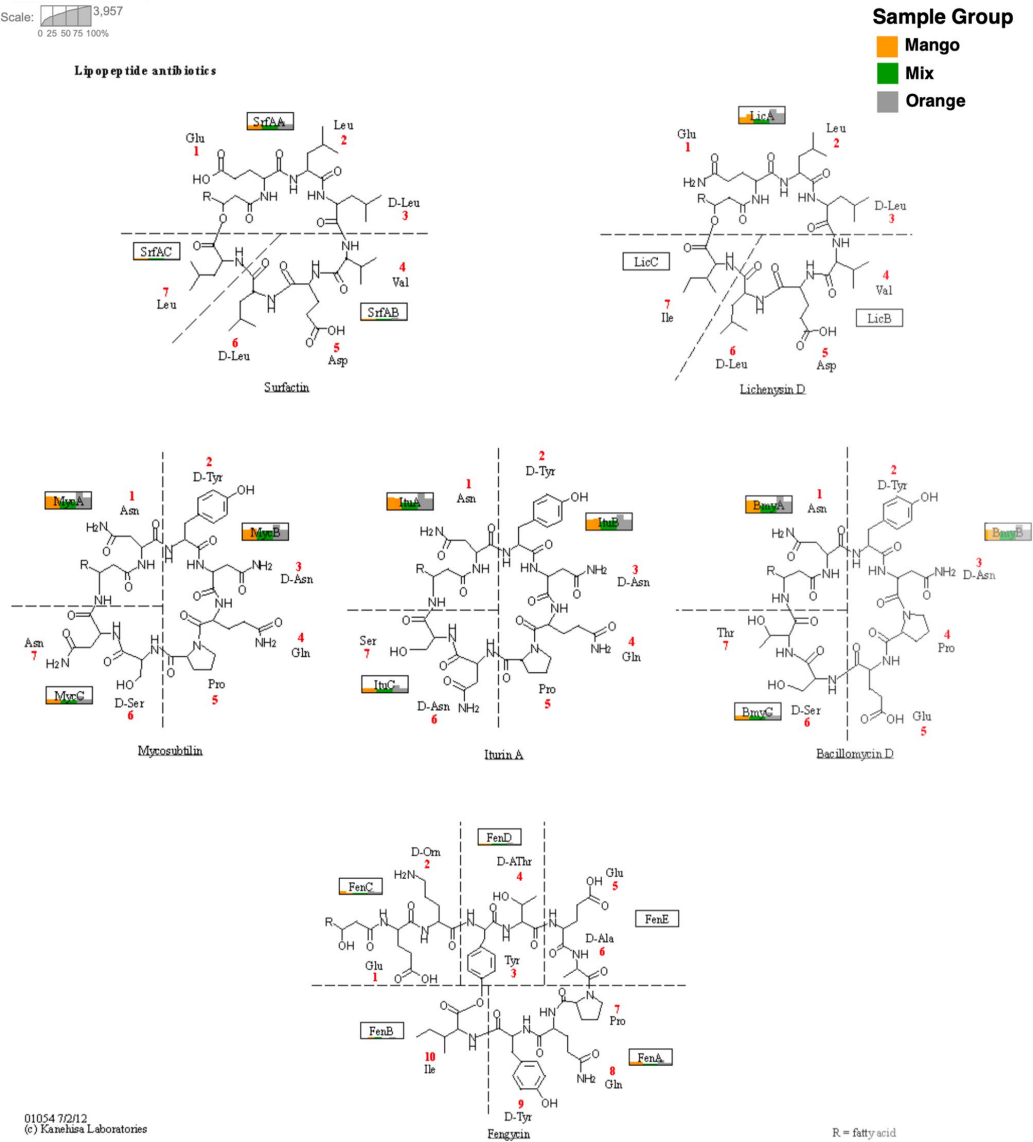
For each of the six fruit-based samples, we indicate the number of reads assigned to the different genes involved in non-ribosomal synthesis. The illustration is based on KEGG pathway ko01054 “Nonribosomal peptide structures”^24^. Each gene is represented by a rectangle and inside each rectangle we use a bar chart to indicate the number of reads assigned from each of the six samples, namely Mango-1 and Mango-2 (yellow), Mix-1 and Mix2 (green) and Orange-1 and Orange-2 (gray).

The results of the BioSurfDB runs of the DIAMOND+MEGAN pipeline were loaded into MEGAN to obtain an overview of the functional potential present in the samples and to allow an assessment of the relative abundances of genes related to biosynthesis of biosurfactants (Fig. 2). Strikingly, more than 60% of all reads that map to a Surfactant class were assigned to a biosynthesis-of-lipopeptide biosurfactant subclass. In addition, a fair number of reads were aligned to genes crucial for synthesizing non-ribosomal lipopeptide structures, namely surfactin, mycosubtilin, iturin A, lichenysin D, bacillomycin D and fengycin (Fig. 3).

**Figure 4:**
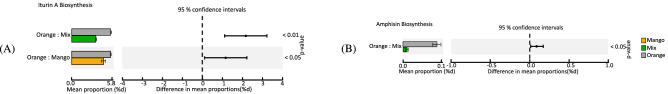
Comparison of functional profiles of fruit samples. Here we only show those biosurfactant types whose mean proportions of assigned reads showed significant differences between samples (p < 0.05, Tukey’s adjustment), computed using STAMP^31^.

In more detail, the most abundant surfactant-related proteins present in the samples were associated with the biosynthesis of pultisolvins followed by trehalolipids, mycosubtilin and iturin A. Trehalolipids are classified as glycolipids while putisolvins, mycosubtilin and iturin A are considered lipopeptides. Significant differences were observed only in the lipopeptide biosynthesis category: iturin A biosynthesis (Fig. 4a) was more abundant in Orange samples than Mix and Mango samples and amphisin biosynthesis (Fig. 4b) was more abundant in Orange samples than in Mix samples.

**Figure 5:**
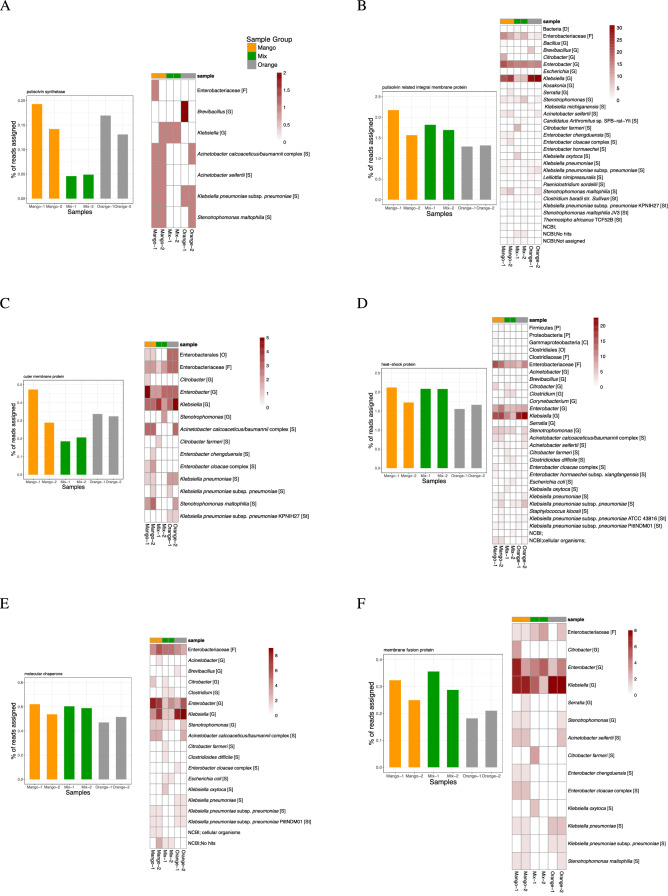
For six different genes involved in putisolvins biosynthesis, under (**A**–**F**), we report the percentage of reads assigned to the gene, and we also show a heatmap indicating how many contigs obtained by gene-centric assembly were assigned to certain taxa, based on their alignment against the NCBI-nt database.

To address the question of which taxa contain the detected biosurfactant genes, we applied gene-centric assembly^29^ to reads assigned to a number of such genes and then determined the taxonomic assignment of the resulting contigs. For a cluster of genes involved in putisolvins production, the resulting contigs were mainly assigned to genera in the above described common core of genera found in the fruit samples. The class Gammaproteobacteria, the family Enterobacteriaceae, and their representatives *Klebsiella* sp. and *Enterobacter* sp. (Fig. 5) were the most assigned to. At a lower abundance, *Acinetobacter* sp. and *Stenotrophomonas* sp. were also assigned to.

The taxonomic assignments of contigs obtained from genes involved in the synthesis of mycosubtilin, iturin A and lichenysin (Fig. 3) were mainly to classes and families associated with the mentioned common core of genera. The genera *Brevibacillus*, *Klebsiella* and *Enterobacter* received the highest assignments (Figure S2).

These results suggest that all six fruit-waste samples have a similar functional profile related to biosurfactant biosynthesis and all samples appear to have a significant potential for the production of biosurfactants.

## Discussion

Our analysis suggests that the microbiota of the six investigated fruit samples share a common core of Gammaproteobacteria and that Enterobacteriaceae is the most abundant family. This is compatible with previous studies that indicate that Enterobacteriaceae are predominant in plant-associated microbiomes^32,33^. *Enterobacter* and *Klebsiella* are endophytic bacteria that are involved in plant survival, production of toxins, antimicrobial compounds, and contribute to plant-growth promotion^34^. Fruit microbiota are diverse and their composition are influenced by management practices, pesticide use, external factors, growing season, etc.^35^. Several genera of Proteobacteria are considered efficient oil-degrading and biosurfactant-producing microorganisms, including *Pseudomonas*, *Streptomyces*, *Enterobacter*, *Acinetobacter*, *Escherichia*, *Klebsiella*, and *Stenotrophomonas*^3,36–40^. Some of these appear as members of the microbial common core presented here.

The results summarized in Figs. 2 and 5 suggest that the functional profiles of the microbiota present in the six fruit samples are quite similar to each other and have the potential for biosurfactant biosynthesis, especially of lipopeptides. Lipopeptides biosurfactants are considered potent antimicrobials that are capable of disrupting the cell membrane^41^. In addition, they also exhibit antitumor, immunomodulatory, emulsifying activities and their production is also closely related to motility and biofilm formation or inhibition^22,42,43^. These biosurfactants are synthesized by non-ribosomal peptide synthases (NRPSs). They have diverse structures, and nutritional parameters influence their composition^44,45^. The high occurrence of genes related to the biosynthesis of lipopeptide biosurfactants may be due to the fact that some endophytic bacteria produce such antimicrobial molecules to help protect plants against pathogens^45,46^.

Previous studies have reported that putisolvins biosurfactants are associated with motility, the dispersion of naphthalene and phenanthrene crystals and also with breaking or inhibiting biofilms^47^. Their production depends on several regulatory genes such as the heat shock system DnaK and the two component system GacA/GacS^48^. Previously, their production has been attributed to the *Pseudomonas putida* species^47,48^, whereas our study now links them to *Klebsiella*, *Enterobacter* and *Acinetobacter*, as well. This suggests the presence of certain regulatory genes for secondary metabolites that are common to gram-negative bacteria^48^. The fact that these bacteria possess genes involved in the biosynthesis of putisolvins may direct studies on the production of these molecules and their applicability in biofilm removal.

Another biosurfactant present is iturin A^1,46^. The genera *Brevibacillus*, *Klebsiella* and *Enterobacter* have the highest number of assignments to genes related to the biosynthesis of members of the iturins family. In addition, all samples have reads that align to genes involved in the synthesis of molecules that are related to surfactin, lichenysin, mycosubitilin, iturin A and bacillomycin, respectively. This suggests that fruit-autochthonous microbiota are a promising source for genes for producing these molecules.

Biosurfactants mainly arise in response to environmental conditions imposed on the microorganism, acting in physiological functions such as motility, protection from toxins, adhesion to substrates and cell interactions^49^. The microbiota of hydrocarbon-contaminated sites have been widely studied, isolated and used in the production of biosurfactants, due to the ability of these microorganisms to utilize hydrophobic carbon sources, and thus play a role in bioremediation. Our work provides a new perspective for prospecting for biosurfactant-producing microorganisms, as it suggests that fruit-waste samples are a promising source of bacteria capable of producing biosurfactants, mainly lipopeptides, which may have application in several industrial sectors. Further, isolation of culture-dependent strains from these fruit residues might be usable in fermentative processes and we intend to explore this further.

Our analysis determined similar profiles directed towards biosurfactant biosynthesis. The samples show high reads counts for biosurfactant production, mainly lipopeptides, a potential source of novel antibiotic and antifungal molecules. Furthermore, in line with the result of taxonomic analysis, the results of the gene-centric analysis showed that the *common core* found in the samples is directly related to the genes of interest.

The BiosurfDB domain-specific database is an essential resource for detecting the presence of genes associated biosurfactant biosynthesis. Integration of the BiosurfDB classification into our interactive metagenome analysis tool allows the user to interactively explore and compare the reads assigned to biosurfactant genes. While the putisolvins genes detected in our samples are represented in BioSurfDB by reference genes obtained from *Pseudomonas* genomes, gene-centric assembly and DNA alignment to the NCBI-nt database clearly show that the organisms carrying these genes in the six samples are not *Pseudomonas*, but rather belong to the genera *Klebsiella*, *Enterobacter* and *Acinetobacter*.

## Methods

### Sampling and autochthonous fermentation

Fruit residues were collected from open fairs in the city of Sorocaba, São Paulo, Brazil. The fruit residues were separated into three distinct samples: orange bagasse, mango residue and mixed fruit residue (using a mixture of fruits that includes papaya, pear, avocado, grapes, guava and banana residues). Each fruit sample was crushed and blended before use. Subsequently, the samples were subjected to autochthonous fermentation in order to promote the growth of the associated microbiota of the residues.

For the autochthonous fermentation, 12.5 g of each fruit sample was added separately into reagent bottles containing 500 mL of Luria-Bertami (LB) medium for enrichment. The autochthonous fermentation occurred for 5 days at 32$^\circ$C and 150 rpm, in duplicates, totaling six batch reactors. Later, the composition of microbiota obtained was analyzed through metagenome sequencing.

### DNA extraction and metagenome sequencing

DNA from each autochthonous fermentation was extracted and purified using the PowerSoil DNA kit (MoBio Laboratories, Inc., Carlsbad, CA, USA), following the manufacturer’s instructions. DNA concentration and quality were estimated using a ND-2000 spectrophotometer (Nanodrop Inc, Wilmington, DE), using a ratio of 260/280 nm > 1.8. For each extraction, the DNA was sequenced in a $2 \times 150$ bp format using a Illumina HiSeq at the Animal Biotechnology Laboratory, Department of Animal Science (ESALQ/USP, Piracicaba, São Paulo, Brazil) according to the manufacturer’s guidelines. This resulted in six datasets, which we will refer to as Orange-1, Orange-2, Mango-1, Mango-2, Mix-1 and Mix-2. These contain very similar numbers of reads, namely $23\,407\,430$, $22\,989\,500$, $24\,136\,468$, $24\,072\,864$, $21\,872\,692$, and 23 704 278, respectively.

All sequencing reads were submitted to the European Nucleotide Archive under project accession PRJEB47062 and sample accessions ERS7265231 (Mango-1), ERS7265232 (Mango-2), ERS7265233 (Mix-1), ERS7265234 (Mix-2), ERS7265235 (Orange-1) and ERS7265236 (Orange-2).

### BioSurfDB representation in MEGAN

The MEGAN software allows incorporation of additional classifications. Here we describe how to add a new functional classification to MEGAN that represents data provided by the BioSurfDB^28^ database, which is focused on biosurfactants and biodegradation. Using the URLs https://www.biosurfdb.org/api/get/pathway, https://www.biosurfdb.org/api/get/protein and https://www.biosurfdb.org/api/get/organism_pathway, we downloaded three files in JSON format from BioSurfDB, which we called pathway.json, protein.json and organism_pathway.json, respectively.

The first file, pathway.json, contains information about pathways. We parsed it to create the basic hierarchical tree representation of entities in the BioSurfDB classification. This representation uses integer identifiers for all nodes in the tree and a separate mapping of those identifiers to names. We provide the tree representation and the mapping in two files called biosurfdb.tre and biosurfdb.map, respectively.

The second downloaded file, protein.json, provides all protein accessions and sequences that occur in the classification. We provide these in a fasta file called biosurfdb.fasta. Finally, the third downloaded file, organism_pathway.json, was used to place the proteins into the tree representation. As the number of proteins is small ($\approx$ 6,000), their accessions were also added to the tree as leaves. The downloaded file was also used to create a mapping of protein accessions to the integer node identifiers. We provide this mapping as the file acc2biosurfdb.map.

As mentioned, MEGAN allows the user to incorporate new classifications into the software. This requires that one prepares two files that describe the hierarchical structure of the classification, such as the two files biosurfdb.tre and biosurfdb.map described above. Use the Edit-Preferences-Add Classification... menu item to select the tree file so as to add the new classification to MEGAN. The program will provide a viewer for the classification and will integrate it into all menus, toolbars and dialogs.

To enable MEGAN to assign reads to a new classification, during the meganization or import of an alignment file, the user must specify an appropriate mapping file that maps reference sequence accessions to entities in the classification. In the case of alignment against the BioSurfDB sequences in biosurfdb.fasta, the appropriate mapping file is acc2biosurfdb.map.

### DIAMOND+MEGAN analysis

All six samples were first aligned against the NCBI-nr database^27^ (downloaded from ftp://ftp.ncbi.nih.gov/blast/db/FASTA/nr.gz in January 2021) using DIAMOND^50^ (version 2.0.0, default alignment options). The resulting DAA files were “meganized”, i.e. subjected to taxonomic and functional binning, using MEGAN^26^ (version 6.21.2, default options). We will refer to this DIAMOND+MEGAN analysis as an NCBI-nr run.

We also aligned all six datasets against the BioSurfDB reference file using DIAMOND (default alignment options), The resulting DAA files were “meganized” with the help of the BioSurfDB-specific mapping file acc2biosurf.map. We will refer to this DIAMOND+MEGAN analysis as a BioSurfDB run.

Taxonomic and functional profiles were exported from MEGAN and statistical analysis was performed using STAMP^31^. In more detail, analysis of variance (ANOVA) were conducted to compare these three groups of functional profiles and *post hoc* tests by Tukey’s adjustment were examined to determine the significant differences between samples. Unclassified reads were removed from the analysis and results with $p< 0.05$ (corrected *p*-value) were considered significant.

To determine the genus-level taxonomic content of the samples, we employed *taxonomic projection* as implemented in MEGAN. In this calculation, all reads assigned at a taxonomic rank that is more specific than genus are “projected up” to the appropriate genus, whereas all reads that are assigned at a higher taxonomic rank are “projected down” onto the subsequent genera, in proportion to the number of reads assigned to each such genus.

### Gene-centric assembly

MEGAN provides an algorithm for assembling all reads that align to a specific class of reference sequences, using protein-alignment-guided assembly, as described in^29^. For each of the six samples, we applied this algorithm (default parameters) to genes associated with Surfactant classes in the BioSurfDB classification.

For the Putisolvins Biosynthesis class we assembled the genes for heat-shock protein, membrane fusion protein, molecular chaperone, outer membrane protein, putisolvin related integral membrane protein, and putisolvin synthetase (see Table 3). For the classes Iturin A Biosynthesis, Lichenysin Biosynthesis and Mycosubtilin Biosynthesis, we assembled the genes for Iturin A synthetase C, lichenysin synthetase A, and both mycosubtilin synthase subunit A and Mycosubtilin synthase subunit B, respectively.

**Table 3:** Basic statistics for the gene-centric assembly of surfactant-related genes. For each of six genes associated with the class Putisolvins Biosynthesis, we report the number of contigs, their minimum, mean and maximum length and their average coverage. The values reported here are for the Mango-1 sample, the values for the other samples are similar and are reported in the Supplement.

Gene name	Number of contigs	Min.	Mean length	Max.	Average coverage
Heat-shock protein	64	201	575	1572	7.2
Membrane fusion protein	26	207	257	330	5.0
Molecular chaperone	26	225	390	528	8.0
Putisolvin related integral membrane protein	84	201	377	750	6.4
Putisolvin synthetase	5	201	245	333	4.3
Outer membrane protein	19	204	254	519	6.1

The resulting contigs were aligned against the NCBI-nt database (downloaded October, 2021) using BLASTN^30^ (default parameters) and the results were then imported into MEGAN so as to perform taxonomic analysis (default parameters, using the megan-nucl-Jan2021.db mapping file). The heatmaps in Fig. 5 (and in supplementary Figure S3) are based on the number of contigs assigned to each taxon, for a given gene and for each sample.

## Supplementary Information

Supplementary Information.

## Data Availability

The six sequences are available from the European Nucleotide Archive under project accession PRJEB47062 and sample accessions ERS7265231 (Mango-1), ERS7265232 (Mango-2), ERS7265233 (Mix-1), ERS7265234 (Mix-2), ERS7265235 (Orange-1) and ERS7265236 (Orange-2). The BioSurfDB extension for MEGAN is available here: https://software-ab.informatik.uni-tuebingen.de/download/megan6/biosurfdb.zip. The MEGAN files for all six samples, and the results of gene-centric assembly, are available here: https://software-ab.informatik.uni-tuebingen.de/download/public/surfactant.
