# Supplementary material for: Interactive analysis of biosurfactants in fruit-waste fermentation samples using BioSurfDB and MEGAN
Source: Sci Rep. 2022 May 11;12:7769. doi: 10.1038/s41598-022-11753-0 (PMC9095615; doi:10.1038/s41598-022-11753-0)
Supplement: Supplementary file 1 — Supplementary Information. [file 41598_2022_11753_MOESM1_ESM.pdf]

Supplementary Data

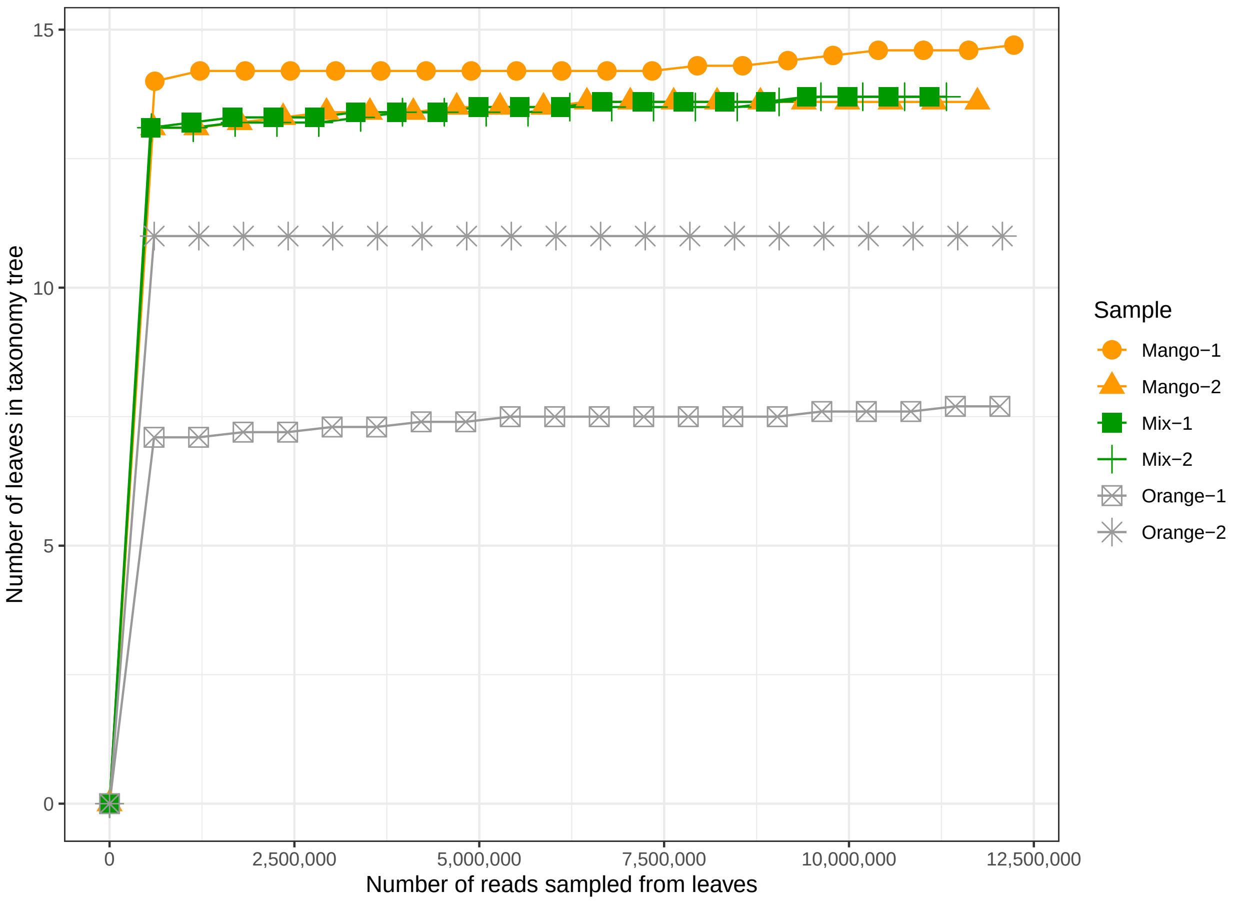

Figure S1: Rarefaction curves for six metagenome sequence data showing genera diversity as a function of number of reads.

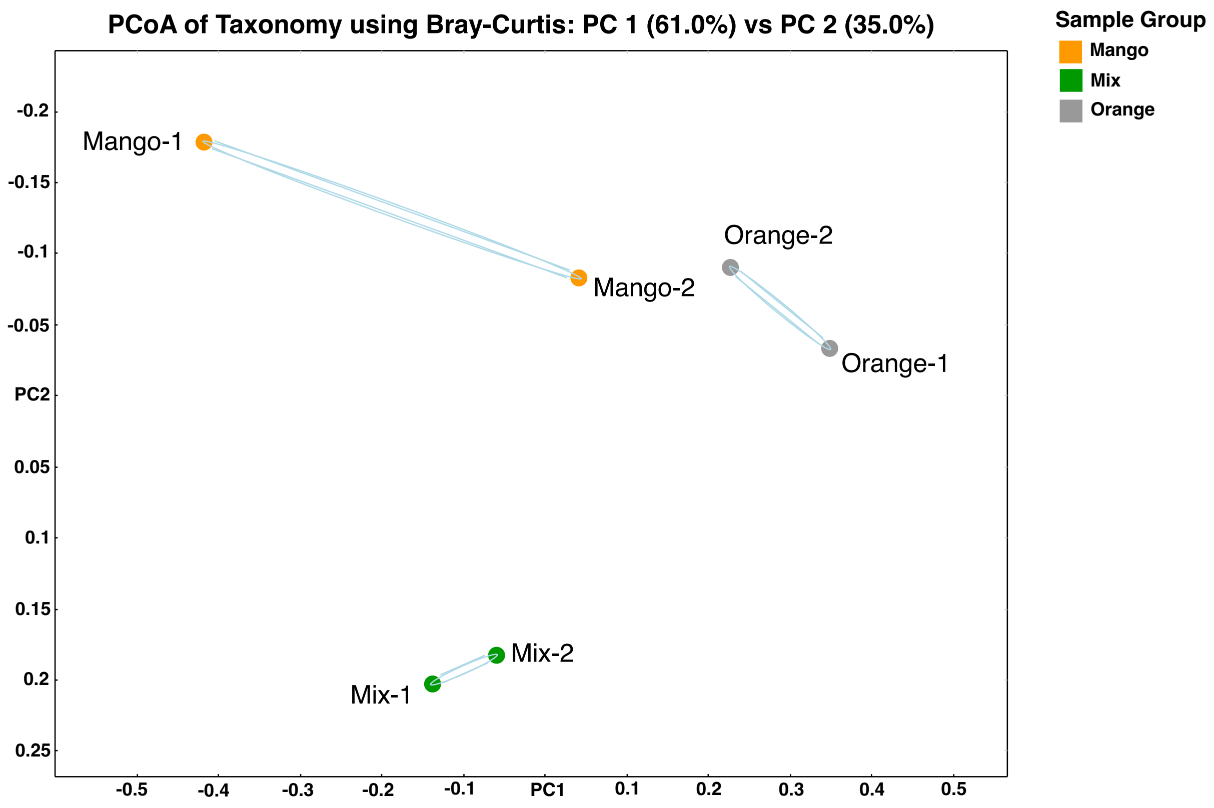

Figure S2: Analysis of β-diversity for six metagenome sequence dataset. Bray-Curtis distances where calculated from normalized genus-level counts and subjected to a principal coordinates analysis (PCoA). Here we plot the two first principal coordinates.

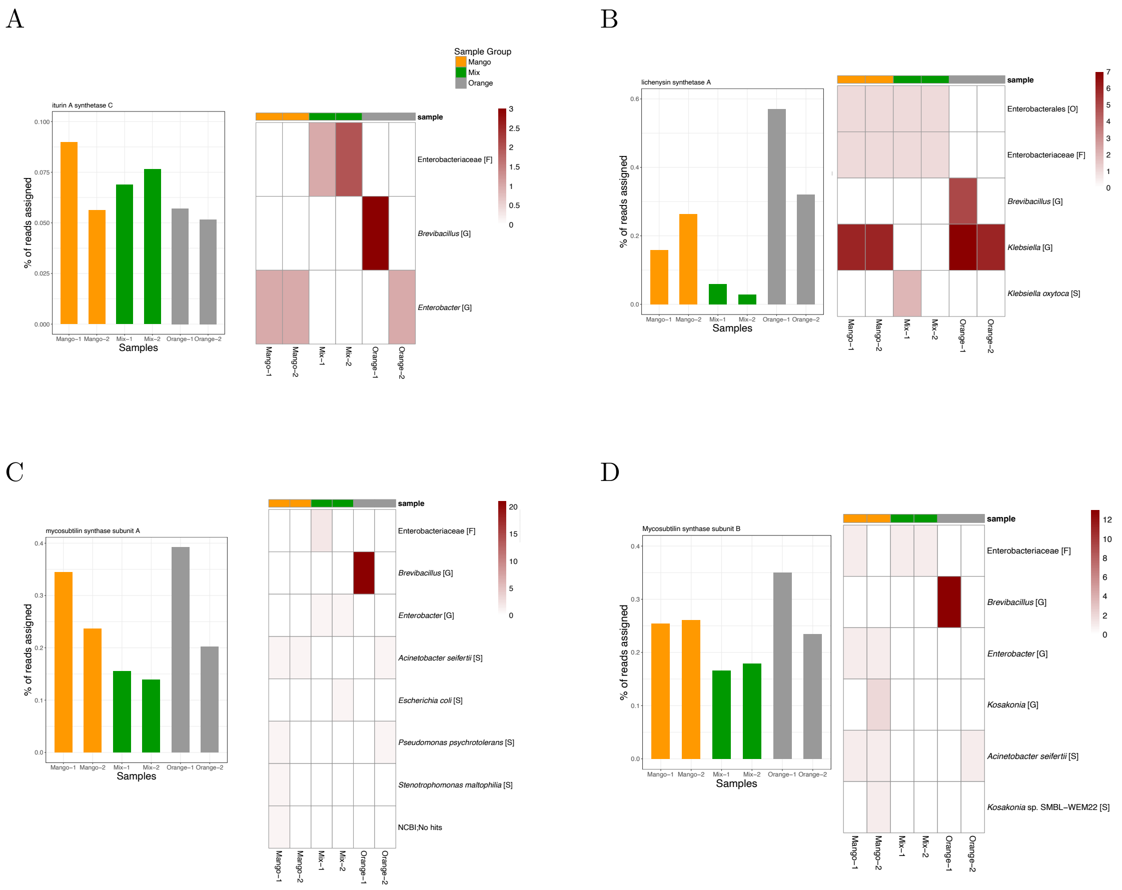

Figure S3: For one gene involved in iturin A biosynthesis, one in lichenysin biosynthesis and two in mycosubtilin biosynthesis, under (A)-(D), respectively, we report the percentage of reads assigned to the gene, and also show a heatmap indicating how many contigs obtained by gene-centric assembly where assigned to certain taxa, based on their alignment against the NCBI-nt database.

Table S1: Basic statistics for the gene-centric assembly of surfactant-related genes. For each of six genes associated with the class Putisolvins Biosynthesis, we report the number of contigs, their minimum, mean and maximum length and their average coverage, based on the separate assembly of all six samples.

Gene name	Number of contigs	Min.	Mean Length	Max.	Average coverage
**Mango-2**					
Heat-shock protein	68	201	561.2	1110	6.5
Membrane fusion protein	21	216	264.1	321	5.1
Molecular chaperone	32	207	359.4	528	6.7
Outer membrane protein	19	204	258.9	519	6.2
Putisolvin related integral membrane protein	78	201	387.3	777	5.9
Putisolvin synthetase	5	210	276.6	333	3.8
**Mix-1**					
Heat-shock protein	43	201	518.7	1137	8.1
Membrane fusion protein	16	201	260.2	318	6.4
Molecular chaperone	18	219	347.7	522	8.9
Outer membrane protein	8	234	250.9	264	7
Putisolvin related integral membrane protein	53	201	369.2	783	6.7
Putisolvin synthetase	1	309	309	309	3.8
**Mix-2**					
Heat-shock protein	36	201	505.2	1125	8.4
Membrane fusion protein	10	225	269.4	318	6.7
Molecular chaperone	15	204	368.4	528	9.2
Outer membrane protein	9	210	241	264	6.4
Putisolvin related integral membrane protein	51	201	370.1	777	6.2
Putisolvin synthetase	1	333	333	333	4.4
**Orange-1**					
Heat-shock protein	39	204	596.2	1155	7
Membrane fusion protein	13	225	269.5	321	5.3
Molecular chaperone	20	237	360.4	528	7.2
Outer membrane protein	16	201	232.5	264	6.3
Putisolvin related integral membrane protein	57	201	360.5	777	5.4
Putisolvin synthetase	3	213	258	333	3.4
**Orange-2**					
Heat-shock protein	61	201	564.6	1137	6.6
Membrane fusion protein	21	216	259.1	327	5.2
Molecular chaperone	26	225	376.7	528	7.2
Outer membrane protein	22	204	255.4	519	5.5
Putisolvin related integral membrane protein	65	201	408	777	5.8
Putisolvin synthetase	3	207	270	333	4.1

Table S2: Basic statistics for the gene-centric assembly of surfactant-related genes. For one gene involved in iturin A biosynthesis, one in lichenysin biosynthesis and two in mycosubtilin biosynthesis, we report the number of contigs, their minimum, mean and maximum length and their average coverage, based on the separate assembly of all six samples.

Gene name	Number of contigs	Min.	Mean Length	Max.	Average coverage
**Mango-1**					
Iturin A synthetase C	1	213	213	213	4.9
Lichenysin synthetase A	8	246	310.1	429	7.3
mycosubtilin synthase subunit A	4	201	268.5	450	5.9
Mycosubtilin synthase subunit B	3	201	242	309	4.1
**Mango-2**					
Iturin A synthetase C	1	207	207	207	4.3
Lichenysin synthetase A	8	246	325.1	438	9.1
mycosubtilin synthase subunit A	1	450	450	450	6.9
Mycosubtilin synthase subunit B	5	216	262.8	345	4.2
**Mix-1**					
Iturin A synthetase C	1	216	216	216	7.6
Lichenysin synthetase A	4	315	326.2	336	5.8
mycosubtilin synthase subunit A	3	204	225	258	3.3
Mycosubtilin synthase subunit B	1	216	216	216	4.7
**Mix-2**					
Iturin A synthetase C	2	216	216	216	7.2
Lichenysin synthetase A	2	318	327	336	5.9
mycosubtilin synthase subunit A	2	204	208.5	213	3.5
Mycosubtilin synthase subunit B	1	216	216	216	6.9
**Orange-1**					
Iturin A synthetase C	3	204	207	213	2.6
Lichenysin synthetase A	12	219	306	444	6.4
mycosubtilin synthase subunit A	21	204	780.4	2820	3.3
Mycosubtilin synthase subunit B	13	201	250.2	276	3
**Orange-2**					
Iturin A synthetase C	1	207	207	207	2.1
Lichenysin synthetase A	6	246	315	444	9.4
mycosubtilin synthase subunit A	2	204	327	450	5
Mycosubtilin synthase subunit B	1	306	306	306	4.3
